# The Synthetic Flavonoid Hidrosmin Improves Endothelial Dysfunction and Atherosclerotic Lesions in Diabetic Mice

**DOI:** 10.3390/antiox11122499

**Published:** 2022-12-19

**Authors:** Luna Jiménez-Castilla, Lucas Opazo-Ríos, Gema Marin-Royo, Macarena Orejudo, Raquel Rodrigues-Diez, Constanza Ballesteros-Martínez, Manuel Soto-Catalán, Teresa Caro-Ordieres, Inés Artaiz, Tatiana Suarez-Cortés, Arturo Zazpe, Gonzalo Hernández, Marcelino Cortés, José Tuñón, Ana M. Briones, Jesús Egido, Carmen Gómez-Guerrero

**Affiliations:** 1 Renal, Vascular and Diabetes Research Laboratory, IIS-Fundación Jiménez Díaz, Universidad Autónoma de Madrid, 28040 Madrid, Spain; 2 Spanish Biomedical Research Centre in Diabetes and Associated Metabolic Disorders (CIBERDEM), 28029 Madrid, Spain; 3 Facultad de Ciencias de la Salud, Universidad de Las Américas, Concepción-Talcahuano 4301099, Chile; 4 Departamento de Farmacología, Universidad Autónoma de Madrid, Instituto de Investigación Hospital La Paz, 28029 Madrid, Spain; 5 Centro de Investigación Biomédica en Red de Enfermedades Cardiovasculares (CIBERCV), 28029 Madrid, Spain; 6 Department of Physiology, School of Medicine, Universidad Complutense de Madrid, 28040 Madrid, Spain; 7 Department of Research, Development, and Innovation, FAES Farma, 48940 Bilbao, Spain; 8 Department of Cardiology, IIS-Fundación Jiménez Díaz, 28040 Madrid, Spain

**Keywords:** hidrosmin, diabetes, obesity, cardiovascular diseases, atherosclerosis, endothelial dysfunction, inflammation, oxidative stress

## Abstract

In diabetes, chronic hyperglycemia, dyslipidemia, inflammation and oxidative stress contribute to the progression of macro/microvascular complications. Recently, benefits of the use of flavonoids in these conditions have been established. This study investigates, in two different mouse models of diabetes, the vasculoprotective effects of the synthetic flavonoid hidrosmin on endothelial dysfunction and atherogenesis. In a type 2 diabetes model of leptin-receptor-deficient (db/db) mice, orally administered hidrosmin (600 mg/kg/day) for 16 weeks markedly improved vascular function in aorta and mesenteric arteries without affecting vascular structural properties, as assessed by wire and pressure myography. In streptozotocin-induced type 1 diabetic apolipoprotein E-deficient mice, hidrosmin treatment for 7 weeks reduced atherosclerotic plaque size and lipid content; increased markers of plaque stability; and decreased markers of inflammation, senescence and oxidative stress in aorta. Hidrosmin showed cardiovascular safety, as neither functional nor structural abnormalities were noted in diabetic hearts. Ex vivo, hidrosmin induced vascular relaxation that was blocked by nitric oxide synthase (NOS) inhibition. In vitro, hidrosmin stimulated endothelial NOS activity and NO production and downregulated hyperglycemia-induced inflammatory and oxidant genes in vascular smooth muscle cells. Our results highlight hidrosmin as a potential add-on therapy in the treatment of macrovascular complications of diabetes.

## 1. Introduction

Atherosclerosis and cardiovascular diseases, such as stroke, coronary artery disease and peripheral artery disease, are the leading causes of the morbidity and mortality associated with diabetes mellitus, accounting for 44% and 52% of deaths in patients with type 1 and type 2 diabetes (T1D and T2D) [1,2]. Additional risk factors often seen in patients with diabetes, such as hypertension, obesity, sedentary behavior, dyslipidemia and smoking, can directly or indirectly affect the structure and function of the vascular wall and accelerate atherosclerosis and diabetic cardiovascular complications [1,3,4]. 

Chronic inflammation, oxidative stress and endothelial dysfunction are interrelated events in the pathogenesis of diabetic atherosclerosis [5]. Clinical and experimental evidence indicates that the metabolic abnormalities in diabetes promote endothelial dysfunction, a crucial early step in atherogenesis characterized by impaired endothelium-dependent vasodilation as a result of the imbalance between nitric oxide (NO) production and accumulation of reactive oxidative species [6,7]. In addition, hyperglycemia directly modulates the expression of inflammatory cytokines, redox balance enzymes and vasoactive markers through specific signaling pathways in endothelial cells and vascular smooth muscle cells (VSMC) [8,9,10]. Activation of these processes further enhances vasoconstriction, inflammatory cell recruitment, cell differentiation and proliferation, ultimately leading to the progression and destabilization of atheroma plaques [4,5,11]. 

Current pharmacological therapies in patients with diabetes have effectively controlled hypertension and hypercholesterinemia, limiting the progression of vascular damage [12]. Statins, a group of anti-atherogenic drugs with pleiotropic actions, significantly reduce non-fatal and fatal cardiovascular events and are the primary approach for dyslipidemia in high-risk patients [13]. Despite improvements in the lifestyle modifications of cardiovascular and renal risk factors and the introduction of novel glucose-lowering drugs, patients with diabetes still have unacceptably high residual risk for vascular complications.

Since inflammation plays an important role in the development and progression of atherosclerosis, there is a tremendous interest in the evaluation of anti-inflammatory agents as an additional therapy for atherothrombosis prevention and treatment [14], as shown in the recent Canakinumab Anti-inflammatory Thrombosis Outcomes Study (CANTOS) trial examining the use of anti-interleukin-1β therapy in patients with atherosclerotic disease [15] and in the colchicine trial [16]. Recently approved antihyperglycemic drug, such as dipeptidyl peptidase-4 inhibitors, can also limit inflammation and atherosclerosis in T2D patients [17]. In this way, there is growing evidence that flavonoids usually show antioxidant and anti-inflammatory activities, and some of them show anti-hyperglycemic and anti-hyperlipidemic effects as well [18,19]. These naturally occurring compounds are part of a selective group of secondary metabolites from seeds, fruits, vegetables and plants and have been used for decades in traditional medicine due to their beneficial properties [18]. Recent technological advances in the pharmaceutical industry are optimizing the purity and in vivo stability of these molecules, thereby improving the bioavailability and therapeutic efficacy [20,21]. 

Hidrosmin is a synthetic flavonoid with a simple structure derived from the natural bioflavonoid diosmin; both compounds show venotonic and vasculoprotective properties and are clinically indicated for the symptomatic treatment of chronic venous insufficiency [22,23,24,25]. Systematic reviews conducted by Cochrane Library and American and European Vascular Surgery Clinical Guidelines validated the effectiveness and safety of hidrosmin and diosmin as oral phlebotonic drugs [26,27,28]. Besides the treatment of chronic venous insufficiency, several preclinical studies have established that diosmin-containing medicinal products protect against hepatic, pulmonary, neurological, gastrointestinal, myocardial and renal injuries [23,24,25,29]. In this line, our previous study was the first to demonstrate a favorable effect of hidrosmin on the reduction of renal damage in diabetic mice [30]; however, the mechanisms and actions of the synthetic flavonoid in macrovascular complications of diabetes have not been scrutinized. 

Within this framework, the aim of this study was the preclinical evaluation of the vasculoprotective activity of hidrosmin in two different models of diabetes. We firstly assessed the potential effects of hidrosmin on vascular dysfunction and structure in the leptin-receptor-deficient mouse (db/db) in susceptible diabetes background BKS, an excellent model of diabesity and metabolic syndrome. Since this T2D model does not develop atherosclerotic lesions, we turned our attention to the streptozotocin (STZ)-induced T1D in atherosclerosis-prone mice devoid of apolipoprotein E (ApoE KO), a mouse model combining chronic hyperglycemia and hyperlipidemia that results in accelerated diabetic atherosclerosis with similarities to human lesions [31]. In these animals we studied the potential of hidrosmin to influence vascular inflammation, oxidative stress and plaque development. The in vivo findings were complemented in vitro by using vascular smooth muscle cells (VSMC) and endothelial cells. Finally, we also examined the cardiovascular safety of hidrosmin on mouse diabetic hearts.

## 2. Materials and Methods

### 2.1. Ethics Statement

All the in vivo experimental procedures were conducted under the 3R principle (replacement, refinement and reduction) strictly in accordance with Directive 2010/63/EU of the European Parliament and were approved by the Institutional Animal Care and Use Committee of IIS-Fundación Jimenez Diaz and Comunidad de Madrid (PROEX 217/19). Mice were housed in ventilated cages (2–3 mice per cage) with usual bedding material and environmental enrichment in a conventional temperature-controlled room (20–22 °C) with a 12 h light/dark cycle and free access to water and standard food.

### 2.2. Design of the Experimental Mouse Models of Diabetes

Homozygous C57BLKS (BKS) inbred db/db mice (12-weeks old males; Envigo RMS; Barcelona, Spain) were randomized to receive 600 mg/kg/day hidrosmin (5-O-beta-hydroxyethyl diosmin; FAES Farma, Bilbao, Spain) in drinking water (db/db + H group, *n* = 10) or tap water as vehicle (db/db group, *n* = 10) and were monitored during 16 weeks of treatment. Heterozygous littermates (db/m group, *n* = 10) were used as non-diabetic, non-obese controls.

ApoE KO mice (14–16 weeks old males; Jackson Laboratory, Bar Harbor, ME, USA) were made diabetic by two daily consecutive injections of STZ (125 mg/kg/day, intraperitoneally; S0130, Sigma-Aldrich, St. Louis, MO, USA) dissolved in 10 mmol/L citrate buffer pH 4.5 [32,33]. After 2 weeks, mice with overt diabetes (glucose > 350 mg/dL) were randomized to receive 300 mg/kg/day of hidrosmin in the drinking water (D group, *n* = 11) or tap water (D + H group, *n* = 7) for 7 weeks, based on our previous study [30].

All mice were fed a standard diet and water ad libitum during the experiments and were monitored every 2–3 days at approximately the same time of day for blood glucose (NovaPro glucometer, Nova Biomedical, Waltham, MA, USA), water consumption and body weight. Both tap water and hidrosmin solution were renewed every 2–3 days. At the end of the study, 12 h-fasted mice were anesthetized (100 mg/kg ketamine and 15 mg/kg xylazine) and saline-perfused. After euthanasia, aorta and mesenteric arteries were obtained. Serum levels of total cholesterol, triglycerides, high-density lipoprotein (HDL) cholesterol, low-density lipoprotein (LDL) cholesterol, hepatic transaminases, urea and blood urea nitrogen were determined by an automatic Roche Cobas analyzer at the central laboratories of our institution. Serum levels of 8-hydroxydeoxyguanosine (8OHdG) were determined in duplicate by the ELISA method (SKT-120, StressMarq Biosciences Inc., Victoria, BC, Canada).

### 2.3. Functional and Structural Vascular Studies

Segments of approximately 2 mm in length obtained from the descending thoracic aorta and 1st branches of the mesenteric resistance artery from db/m (*n* = 7), db/db (*n* = 10) and db/db + H (*n* = 8) mouse groups were used to study vascular function in a wire myograph. Both aortic rings and mesenteric arteries were maintained in cold Krebs–Henseleit solution (KHS) (115 mmol/L NaCl, 25 mmol/L NaHCO_3_, 4.7 mmol/L KCl, 1.2 mmol/L MgSO_4_. 7H_2_O, 2.5 mmol/L CaCl_2_, 1.2 mmol/L KH_2_PO_4_, 11.1 mmol/L glucose and 0.01 mmol/L Na_2_-EDTA, pH 7.4) and dissected by removing perivascular adipose tissue. After dissection, vessels were mounted in an isometric myograph (Danish Myo Tech, Aarhus, Denmark). After an equilibration period for 30 min at 37 °C in the organ bath, the arterial diameter for optimal tension development was determined as described [34]. Segment’s viability was then studied by initial exposure to a high-potasium (K^+^) solution (120 mmol/L KCl) in KHS (K^+^-KHS). After 30 min, concentration-response curves to acetylcholine (1 nmol/L–10 μmol/L) were performed to assess endothelium-dependent vasodilator responses. Next, concentration-response curves to phenylephrine (1 nmol/L–10 µmol/L) and the NO donor diethylamine NONOate (DEA-NO) (1 nmol/L to 10 μmol/L) were performed to determine vasoconstrictor- and endothelium-independent vasodilator responses, respectively. For determination of vasodilator responses, vessels were precontracted with phenylephrine (aorta) or the thromboxane A2 receptor agonist U46619 (mesenteric arteries) until approximately 50% K^+^-KHS contraction. Data were recorded and analyzed with LabChart^®^ software v7 (ADInstruments, Oxford, UK). Vasodilator responses were expressed as percentage of the previous tone. Vasoconstrictor responses were expressed as mN/mm. Phenylephrine hydrochloride (Cat-No. P6126) and acetylcholine chloride (Cat-No. A9101), DEA-NO (Cat-No. D5431) were purchased from Sigma-Aldrich and dissolved in distilled water. U46619 was purchased from Cayman Chemical and solved in ethanol.

In another set of experiments, aortic segments from C57BL/6J male mice (*n* = 5) were precontracted with phenylephrine and concentration responses to hidrosmin (0.1 to 1 mmol/L) were assessed in arteries, preincubated or not, with the non-selective NO synthase inhibitor NG-nitro-L-arginine methyl ester (L-NAME) (100 μmol/L, for 30 min). In other segments, hidrosmin (1 mmol/L, 1 h) was added to aortic segments before concentration response curves to phenylephrine.

### 2.4. Pressure Myography Studies

The structural properties of mesenteric arteries from db/db mice were studied with a pressure myograph (Danish Myo Tech). The arteries were mounted between two glass microcannula in KHS without calcium (0Ca^2+^) by omitting calcium and adding 1 mmol/L of Ca^2+^ chelant EGTA and equilibrated at an intraluminal pressure of 45 mmHg at 37 °C. After 30 min of equilibration, a pressure–diameter curve was performed by increasing the intraluminal pressure from 3 to 120 mmHg in 20 mmHg intervals. Each intraluminal pressure was maintained for 2 min, and an image was acquired at the end of that period. From these images, measurements of internal and external diameters (Di and De) were obtained under passive conditions at each intraluminal pressure using ImageJ software. Structural and mechanical parameters were calculated as described [34]. Briefly,

Cross-sectional area = π/4 × (De^2^ − Di^2^).

Wall thickness = (De − Di)/2.

Wall/lumen ratio = (De − Di)/2Di.

### 2.5. Histological and Immunohistochemical Analysis of Atherosclerotic Lesions

To analyze the area and composition of atherosclerotic lesions in diabetic ApoE KO mice, the aortic roots (D group, *n* = 7; D + H group, *n* = 11) were embedded in optimal cutting temperature compound and cryosectioned. Atherosclerotic lesion area (μm^2^) and neutral lipid content were quantified in serial 7 μm aortic sections (covering ≈ 1000 μm from valve leaflets) after oil-red-O (ORO)/hematoxylin staining, and individual maximal values were calculated by averaging 2 to 3 sections [33]. Collagen content was determined by picrosirius red staining. For immunohistochemistry, aortic sections were fixed in acetone and blocked for endogenous peroxidase (3% H_2_O_2_ in methanol, 30 min) and nonspecific binding (8% host serum, 30 min). Slides were incubated overnight at 4 °C with primary antibodies against CD3 (Agilent Cat# A0452, RRID:AB_2335677), CD68 (Abcam Cat# ab53444, RRID:AB_869007) telomerase reverse transcriptase (TERT; Thermo Fisher Scientific Cat# MA5-16034, RRID:AB_11153210) and 8OHdG (Abcam Cat# ab10802, RRID:AB_297482), followed by biotinylated secondary antibodies and avidin–biotin complex reagent (Vector Laboratories). Immunoreactive cells were then visualized by the addition of peroxidase substrates (3,3-diaminobenzidine or 3-amino-9-ethylcarbazole; DAKO) and counterstained with hematoxylin. VSMC content was determined by immunofluorescence with antibody against α-smooth muscle actin (α-SMA-Cy3, Sigma-Aldrich Cat# C6198, RRID:AB_476856) and 4′,6-diamidino-2-phenylindole (DAPI) nuclear counterstain. All of the histological evaluations were conducted in a blinded fashion. Positive staining was quantified in at least two sections per mice using Image Pro-Plus (Media Cybernetics, Bethesda, MD, USA) and expressed as percentage of total plaque area. Plaque complexity was assessed by the Stary score [35] as follows: grade G1, early plaques containing foam cells; grade G2, lesions containing foam cells and few cholesterol clefts; grade G3, lesions containing foam cells and numerous cholesterol clefts; and grade G4, advanced plaques with a large lipid core.

### 2.6. In Vitro Experiments

Primary VSMC were isolated from mouse aorta by enzymatic digestion with collagenase type II (C6885, Sigma-Aldrich) as previously described [32,33]. Cells were maintained in Dulbecco’s Modified Eagle Medium (DMEM, D6546; Sigma-Aldrich) containing 10% fetal bovine serum (FBS), 100 U/mL penicillin, 100 μg/mL streptomycin and 2 mmol/L L-glutamine (Sigma-Aldrich) and were used between the third to sixth passages. VSMC were made quiescent by overnight incubation in FBS-free medium, then pretreated for 90 min with hidrosmin at a range of doses (0.1–1 mmol/L) before stimulation with high-glucose (HG, 30 mmol/L D-Glucose; Sigma-Aldrich) for 24 h, and then were processed for RNA analysis. Cell viability was assessed by the 3-(4,5-dimethylthiazol-2-yl)-2,5-diphenyltetrazolium bromide tetrazolium (MTT) assay.

The human microvascular endothelial cell line (HMEC-1; CRL-3243, ATCC, Manassas, VA, USA) was grown in MCDB 131 medium (10372019; Thermo Fisher Scientific, Waltham, MA, USA) supplemented with 10% FBS, 100 U/mL penicillin, 100 μg/mL streptomycin, 10 mmol/L L-glutamine, 10 ng/mL epidermal growth factor and 1 μg/mL hydrocortisone. HMEC-1 were serum-depleted for 24 h and treated for different times with and without hidrosmin (0.1–1 mmol/L). NO production was assessed by measuring the concentration on stable nitrite and nitrate levels in cell supernatants using a colorimetric assay kit (Cat# 780001; Cayman Chemical, Ann Arbor, MI, USA), and values were normalized to total protein concentration. For Western blot analysis, cells were lysed in cold lysis buffer supplemented with phosphatase and protease inhibitors. Equal amounts of total protein were electrophoresed, transferred to PVDF membranes and blotted with antibodies against phosphorylated eNOS at Ser1177 (eNOS (pS1177); BD Biosciences Cat# 612392, RRID:AB_399750) and total eNOS protein (Cell Signaling Technology Cat# 9572, RRID:AB_329863), followed by peroxidase-conjugated secondary antibodies and chemiluminescence detection. The protein bands were quantified by densitometry, and values of phosphorylated (activated) enzyme were normalized to total protein expression. 

### 2.7. mRNA Expression Analysis

Total RNA from thoracic–abdominal aorta of diabetic ApoE KO mice (D group, *n* = 7; D + H group, *n* = 11) and cultured VSMC (*n* = 3 experiments in duplicate) was extracted with TRI reagent (Life Technologies) and quantified (Nanodrop ND-1000 Spectrophotometer, Wilmington, DE, USA). For each sample, 1.5 μg of total RNA was reversely transcribed into cDNA using High-Capacity cDNA Reverse Transcription Kit (Applied Biosystems, Foster City, CA, USA). Target gene expression (Supplementary Table S1) was analyzed in duplicate by real-time PCR using a 7500 Fast Real-Time PCR system (Applied Biosystems) using TaqMan (Applied Biosystems) or SYBR Green Gene Expression (self-designed) detection assays. Expression levels were normalized to 18S rRNA housekeeping gene. The relative expression was determined using the formula 2^-∆Ct.

### 2.8. Echocardiography Assessment

Transthoracic echocardiography in db/db mice (db/m group, *n* = 8; db/db group, *n* = 8; db/db + H, *n* = 8) was performed blinded using a portable LoGIQ-e ultrasound system (GE Healthcare, Chicago, IL, USA) with a 20 MHz center frequency transducer (L10-22 probe). Initially, anesthesia was induced by putting the mouse in an induction chamber using 3–4% isoflurane and 1 L/min 100% oxygen for 30–45 s. Once the animal lost its righting reflex, it was laid supine on a heated platform with its nose enveloped in a nosecone to keep the mouse anesthetized by 1–2% isoflurane. This protocol was adapted to standardize a range of heart rates needed to perform the measurement (400–500 bpm), as well as to provide a pre-established safety state for both the test animal and the cardiologist specialist [36]. Body temperature was maintained with a heated platform. 

The left ventricular (LV) structural and functional parameters were calculated from the LV parasternal short-axis M-mode view, which was recorded at the level of papillary muscles. An M-mode cursor was positioned perpendicular to the anterior and posterior walls, in the middle of the LV for measuring wall thickness and chamber dimensions. LV interventricular septal thickness (IVS), LV internal diameter (LVID) and posterior wall thickness (PW) were measured both end-diastole and end-systole (IVSd, LVIDd, PWd and IVSs, LVIDs, PWs, respectively) from this M-mode view. Image depth, sweep rate and gain settings were used to optimize image quality. Data were averaged based on the measurements of at least three cardiac cycles. Ejection fraction (EF), fractional shortening (FS) and posterior wall thickness (PWT) were calculated using the following formulas:

$$\mathrm{EF}\;(\%)=100\;\times \;\left[{\left({\mathrm{LVIDd}}^{3}-\mathrm{LVIDs}\right)}^{3}/{\mathrm{LVIDd}}^{3}\right]\;\mathrm{FS}\;(\%)=100\;\times \;\left[(\mathrm{LVIDd}-\mathrm{LVIDs})/\mathrm{LVIDd}\right]\;\mathrm{PWT}\;(\%)=100\;\times \;\left[\left(\mathrm{PWs}-\mathrm{PWd}\right)/\mathrm{PWd}\right]$$

### 2.9. Statistical Analysis

Results are shown as individual data and mean ± standard error of the mean (SEM) from separate animals and independent experiments. In the analysis, technical replicates were averaged to provide a single value per each biological replicate. Statistical analyses were performed using GraphPad Prism v7 (GraphPad Software Inc., La Joya, CA, USA). For functional vascular studies, the maximal effect (R_max_) and the pD2 (the negative logarithm of the concentration required to cause 50% of the maximum, EC_50_) were calculated for each concentration–response curve using non-linear regression analysis. Differences across groups were considered significant at *p* < 0.05 using the unpaired Mann–Whitney U-test and Kruskal–Wallis test followed by Dunn’s multiple comparisons test or two-way ANOVA followed by Bonferroni’s post-hoc test, when appropriate.

## 3. Results

### 3.1. Hidrosmin Treatment Improves Endothelial Function in Aorta and Mesenteric Arteries

**Figure 1:**
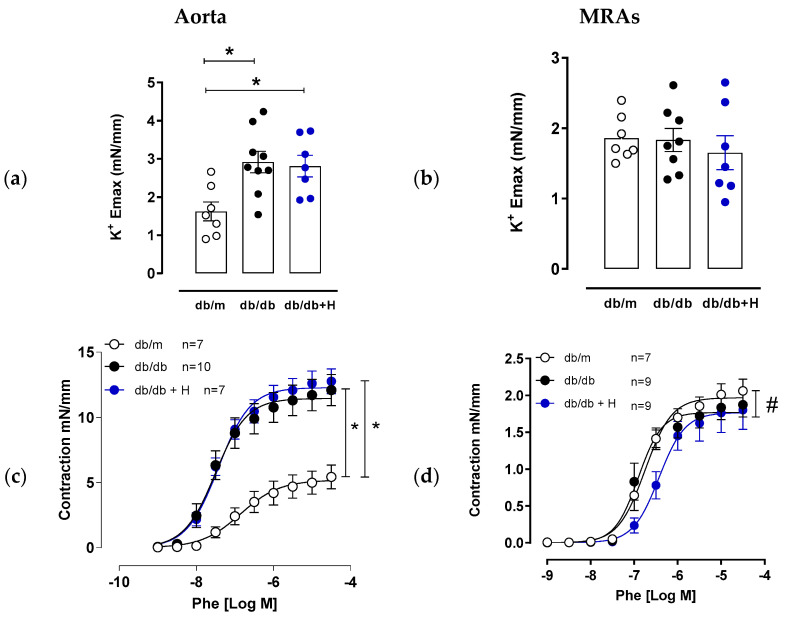

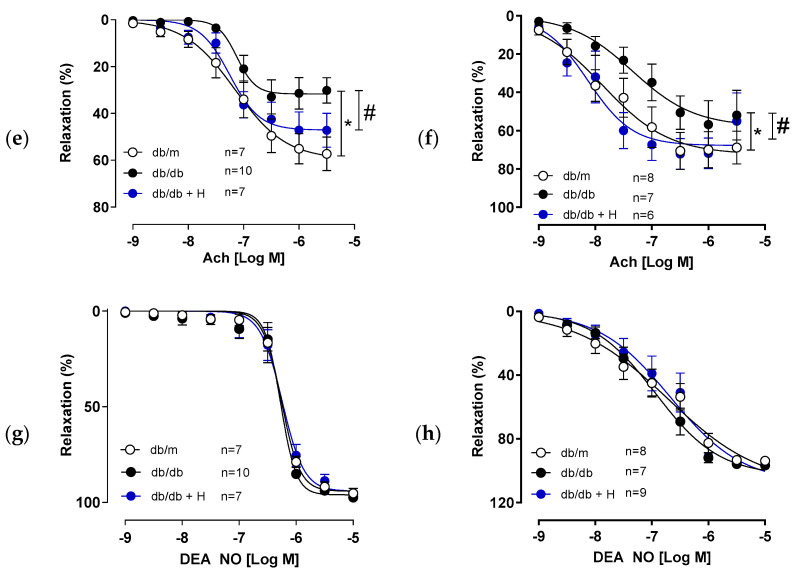
Effect of hidrosmin treatment in vascular function of aorta and mesenteric arteries from db/db mouse model. Maximum responses to high K^+^ concentration in aorta (**a**) and mesenteric resistance arteries (MRAs) (**b**) from non-diabetic (db/m), untreated diabetic (db/db) and hidrosmin-treated diabetic (db/db + H) mice. Graphs represent individual data and the mean ± SEM of each group (db/m, *n* = 7; db/db, *n* = 8–9; db/db + H, *n* = 7) * *p* < 0.05 vs. db/m by Kruskal–Wallis, followed by Dunn’s post-test. (**c**–**h**) Concentration–response curves to phenylephrine (Phe), acetylcholine (Ach) and diethylamine NONOate (DEA-NO) of aortas (**c**,**e**,**g**) and mesenteric arteries (**d**,**f**,**h**). Graphs represent the mean ± SEM of the indicated number of animals per group. * *p* < 0.05 vs. db/m and # *p* < 0.05 vs. db/db by two-way ANOVA followed by Bonferroni’s post-test.

Diabetes is associated with augmented contractile responses to different stimuli, including high extracellular K+ concentration and α-adrenoceptor agonists) in large and small arteries [9,37,38,39]. Our functional studies showed that aorta of db/db mice presented increased contractile response to a high concentration of KCl compared to db/m group and that response was not modified by hidrosmin treatment (Figure 1a). However, in mesenteric resistance arteries, KCl contractile response was similar in the three groups. Conversely, aorta, but not mesenteric arteries from db/db mice, showed an increased contractile response to phenylephrine compared to non-diabetic db/m mice (Figure 1c,d and Supplementary Table S2). Hidrosmin treatment did not modify phenylephrine hypercontraction in aorta of db/db mice and slightly decreased phenylephrine contractile responses in mesenteric arteries (Figure 1c,d and Supplementary Table S2). Noteworthy, aorta and mesenteric arteries from db/db mice showed an impaired endothelium-dependent vasodilator response to acetylcholine that was restored by hidrosmin treatment (Figure 1e,f and Supplementary Table S2). Finally, no differences in vasodilator responses to the NO donor DEA-NO were observed between the groups, either in aortas or in mesenteric arteries (Figure 1g,h and Supplementary Table S2), indicating that the observed effects in endothelial function are not due to changes in VSMC sensitivity to NO. 

**Figure 2:**
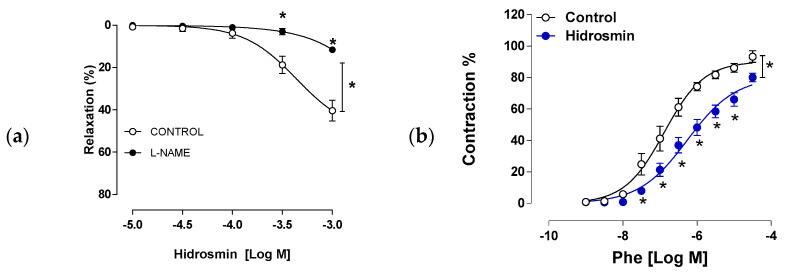
Effects of hidrosmin in vascular function of C57BL6 mouse aorta. (**a**) Concentration–response curves to hidrosmin in the absence (control) and presence of L-NAME. (**b**) Concentration–response curves to phenylephrine (Phe) in the absence (control) and presence of hidrosmin. Graphs represent the mean ± SEM of 4–5 independent experiments. * *p* < 0.05 vs. control by two-way ANOVA, followed by Bonferroni’s post-test.

In another set of experiments, we evaluated the ability of hidrosmin to induce vasodilator responses as well as the potential role of NO. We observed that hidrosmin induced a concentration-dependent vasodilator response in aorta that was partially neutralized by the NOS inhibitor L-NAME (Rmax 40.34 ± 4.9 and 11.54 ± 1.3 for control and L-NAME respectively; *p* = 0.029 by Kruskall–Wallis with Dunn’s post-test) (Figure 2a). Hidrosmin also inhibited contractile response to phenylephrine in mouse aortic rings (Rmax 93.25 ± 3.7 and 79.96 ± 2.7 for control and hidrosmin, respectively; *p* = 0.03 by Kruskall–Wallis with Dunn’s post-test) (Figure 2b). 

### 3.2. Hidrosmin Treatment Does Not Affect Structural Properties of Aorta and Mesenteric Arteries

**Figure 3:**
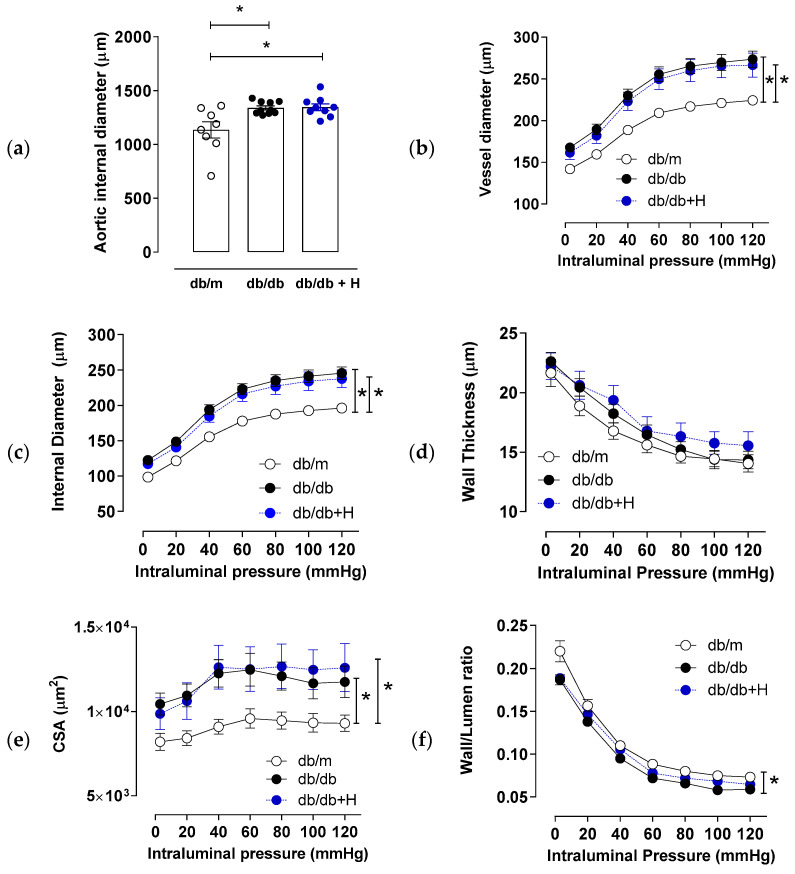
Effect of hidrosmin treatment on structural properties of mesenteric arteries from db/db mouse model. Aorta (**a**) and mesenteric resistance arteries (**b**–**f**) structural parameters in non-diabetic mice (db/m, *n* = 8–6), untreated diabetic mice (db/db, *n* = 10) and hidrosmin-treated diabetic mice (db/db + H, *n* = 8–9). Graphs represent individual values (**a**) and mean ± SEM of each group (**a**–**f**). * *p* < 0.05 vs. db/m by Kruskal–Wallis, followed by Dunn’s post-test (**a**) or two-way ANOVA, followed by Bonferroni’s post-test (**b**–**f**). Abbreviation: CSA, cross-sectional area.

Aortic normalized internal diameter was higher in db/db mice compared to non-diabetic db/m mice, and this was not modified by hidrosmin treatment (Figure 3a). Mesenteric resistance arteries from db/db mice presented hypertrophic outward remodeling with greater internal and external diameter (Figure 3b,c), similar wall thickness (Figure 3d), increased cross sectional area (Figure 3e) and a slight decrease in wall-to-lumen ratio (Figure 3f) compared to db/m group. None of these structural properties were altered by hidrosmin treatment.

In addition, db/db mice showed differences in biochemical parameters compared to the non-diabetic db/m group, but no changes in these metabolic endpoints were observed in the hidrosmin treatment group compared to the untreated diabetic group (Table 1).

### 3.3. Hidrosmin Prevents Atherosclerosis Development and Partially Reverts Features of Plaque Instability, Inflammation and Oxidative Stress in Diabetic Mice

The potential therapeutic role of hidrosmin in atherosclerosis was explored in STZ-induced diabetic ApoE KO mice. There were no statistically significant differences between untreated and hidrosmin-treated groups in terms of body weight and blood glucose at any time point throughout the experiments (Supplementary Figure S1). Treatment improved the survival rate of STZ-induced diabetic mice by 15% (not shown). At the end of the study, hidrosmin-treated mice showed a reduction of triglycerides and total cholesterol levels in comparison to untreated diabetic mice (Table 2).

**Figure 4:**
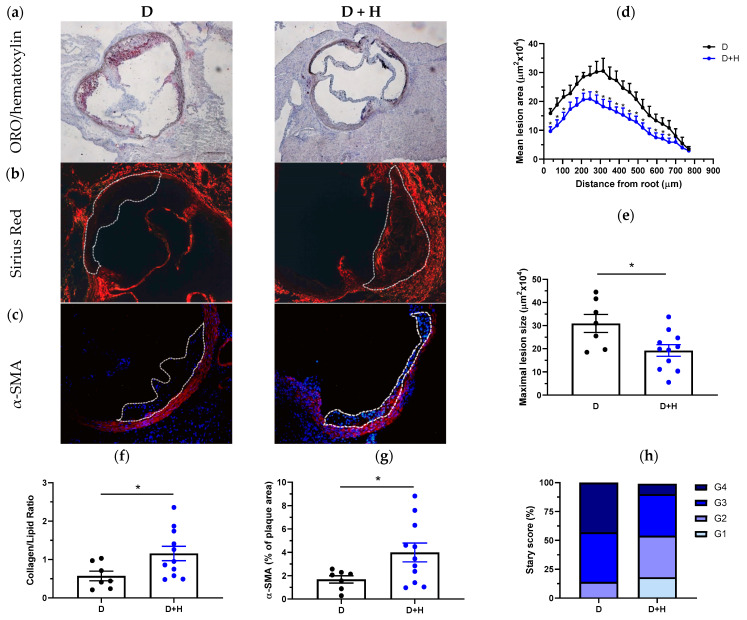
Hidrosmin treatment reduces atherosclerosis development in diabetic ApoE KO mice. Representative images of ORO/hematoxylin staining (**a**), Sirius red collagen staining (**b**) and VSMC immunodetection ((**c**); red, α-SMA; blue, DAPI nuclear staining) in aortic root sections of diabetic mice untreated (D) and treated with hidrosmin (D + H). Magnification ×100 (**a**) and ×200 (**b**,**c**). (**d**) Quantification of the extent of atherosclerotic lesions within the aorta. (**e**) Average of individual maximal lesion size in each group. (**f**) Assessment of collagen-to-lipid ratio. (**g**) Quantification of VSMC content in lesions. (**h**) Classification of mouse atherosclerotic plaques according to the Stary method (grades G1 to G4). Graphs represent individual values and mean ± SEM of each group (D, *n* = 7; D + H, *n* = 11). * *p* < 0.05 vs. D group by Mann–Whitney U test.

Morphometric analysis in serial aortic root sections stained with ORO/hematoxylin (Figure 4a) revealed that hidrosmin treatment markedly reduced the extension of atherosclerotic lesion along the aorta (Figure 4d) and the mean maximal lesion area (% reduction vs. untreated: 38 ± 8, *p* = 0.0168; Figure 4e). We also observed a protective effect of hidrosmin on several histological features of plaque stability. Compared with atherosclerotic lesions from untreated group, hidrosmin-treated mice displayed lower lipid deposition (ORO staining; Figure 4a) and higher collagen content (Sirius red staining; Figure 4b) and therefore a significantly higher collagen-to-lipid ratio (% increase vs. untreated: 201 ± 33, *p* = 0.0376; Figure 4f). We also observed an increased intimal VSMC content (α-SMA staining; Figure 4c) in hidrosmin-treated mice (% increase vs. untreated: 237 ± 48, *p* = 0.0426; Figure 4g), compatible with lower plaque vulnerability. Furthermore, the evaluation of the Stary score showed a remarkable difference between groups. In untreated mice, approximately 40% of plaques were classified as advanced (grade G4), and only 14% were early plaques (grades G1–G2). In contrast, plaques in the hidrosmin-treated group were mostly of grades G2–G3 (72%), while only 9% were advanced plaques (grade G4) (Figure 4h).

**Figure 5:**
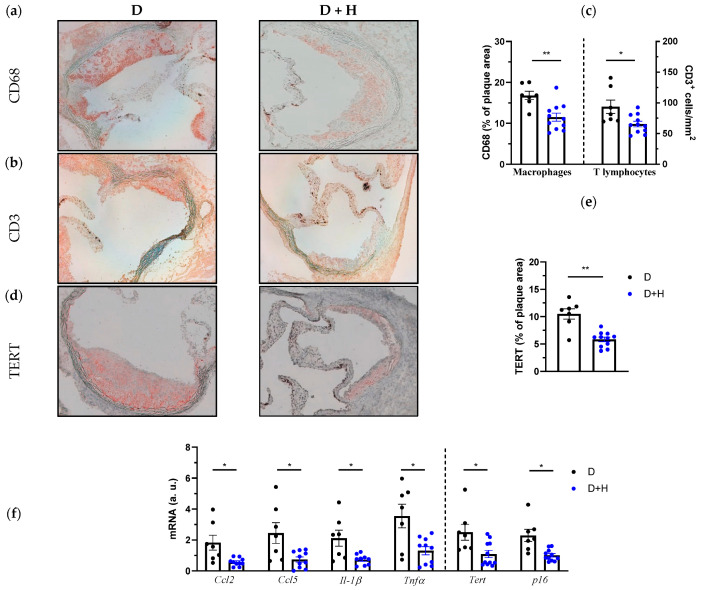

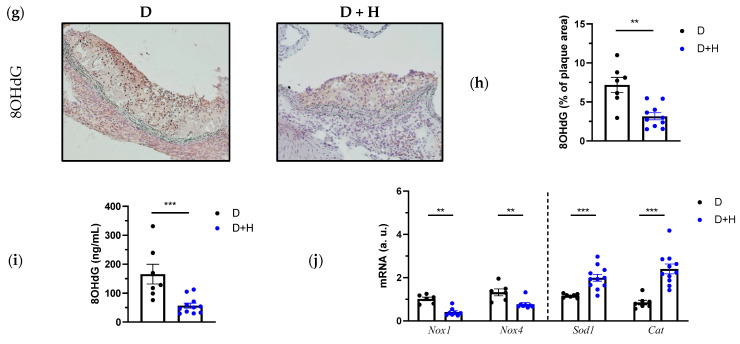
Attenuated inflammation, senescence and oxidative stress in aortic lesions of hidrosmin-treated mice. Representative images (magnification ×200) for CD68+ macrophages (**a**) and CD3+ T cells immunostaining (**b**) in aortic lesions from diabetic ApoE KO mice (untreated, D; hidrosmin treatment, D + H) and quantification (**c**). (**d**) Representative images (magnification ×200) of TERT immunostaining and quantification (**e**). (**f**) Real-time PCR analysis of inflammatory (*Ccl2*, *Ccl5*, *Il-1β* and *Tnfα*) and senescence (*Tert* and *p16*) genes in mouse aorta. Values normalized to 18S rRNA are expressed as arbitrary units (a.u.). (**g**) Representative images (magnification ×200) of 8OHdG immunoperoxidase in mouse aorta and quantification of positive staining (**h**). (**i**) Quantification of 8OHdG levels in serum samples by ELISA method. (**j**) Gene expression analysis of pro-oxidant (*Nox1* and *Nox4*) and antioxidant (*Sod1* and Catalase, *Cat*) genes in mouse aorta. Values normalized to 18S rRNA are expressed as arbitrary units (a.u.). Graphs represent individual values and mean ± SEM of each group (D, *n* = 6–7; D + H, *n* = 8–11). * *p* < 0.05, ** *p* < 0.01 and *** *p* < 0.001 vs. D group by Mann–Whitney U test.

We next sough to investigate the impact of hidrosmin on plaque inflammation, cellular senescence and oxidative stress. Atherosclerotic lesions of hidrosmin-treated mice exhibited a reduced content of CD68+ macrophages and CD3+ T lymphocytes when compared to untreated group (% reduction: 31 ± 6, *p* = 0.0026, and 30 ± 5, *p* = 0.0145, respectively; Figure 5a–c). In addition, real-time PCR analysis revealed a decreased expression of chemokines (CCL2 and CCL5) and cytokines (IL-1β and TNFα) in aortic samples from treated mice (Figure 5f), thus confirming the anti-inflammatory effect of hidrosmin. Treatment also attenuated vascular senescence in diabetic mice, as evidenced by reduced protein and mRNA levels of TERT (Figure 5d–f) and lower mRNA expression of p16 (Figure 5f).

In this study we also determined the vascular and systemic levels of 8OhdG, a sensitive biomarker for oxidative DNA damage. Noteworthy, 8OhdG immunodetection revealed that hidrosmin significantly decreased oxidative stress in diabetic mice, both in atheroma plaques (% reduction vs. untreated: 43 + 8, *p* < 0.01; Figure 5g,h) and in serum samples (Figure 5i). Moreover, hidrosmin therapy partially reversed the aortic expression of redox balance genes by downregulating pro-oxidant enzyme NADPH oxidase (NOX1 and NOX4 isoforms) and upregulating the antioxidant enzymes superoxide dismutase (SOD1) and catalase (Figure 5j).

### 3.4. Effects of Hidrosmin on Cultured Vascular Cells

**Figure 6:**
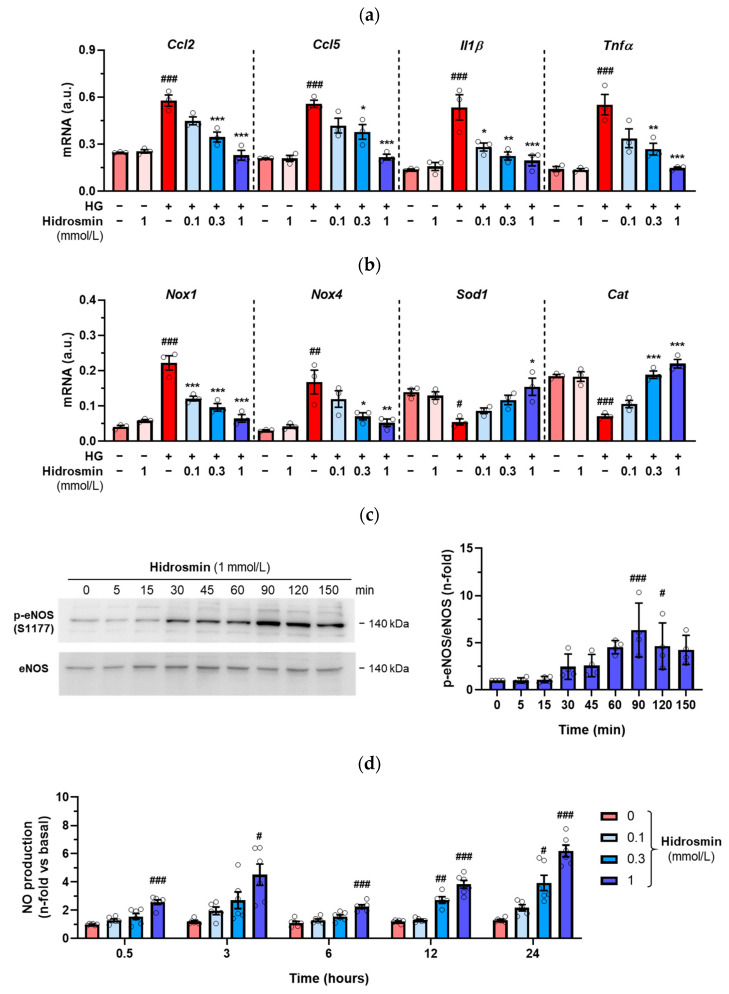
In vitro effects of hidrosmin. Real-time PCR analysis of inflammatory (**a**) and redox balance (**b**) genes in VSMC cultured under basal and high-glucose (HG) conditions without/with different concentrations of hidrosmin. Values normalized to 18S rRNA (*n* = 3 independent experiments performed in duplicate) are expressed as arbitrary units (a.u.). (**c**) Time course of hidrosmin-induced eNOS phosphorylation in HMEC-1 cells. Representative immunoblots of p-eNOS (S1177) and total eNOS (*n* = 4 experiments) and summary of normalized quantifications expressed as fold increases over basal are shown. (**d**) Time- and dose-response curves of hidrosmin-induced NO production in HMEC-1 cells. NO (nitrite + nitrate) levels in cell supernatants were normalized to protein content (*n* = 4–6 experiments). Graphs represent individual values and mean ± SEM. # *p* < 0.05, ## *p* < 0.01 and ### *p* < 0.001 vs. basal; * *p* < 0.05, ** *p* < 0.01 and *** *p* < 0.001 vs. HG.

The in vitro effects of the synthetic flavonoid hidrosmin were investigated in VSMC and endothelial cells, key effector cells in vascular dysfunction that modulate vascular tone, redox balance and inflammatory response to pathological conditions, including diabetes [11]. Firstly, primary mouse VSMC were exposed to high-glucose medium (30 mmol/L glucose) in an attempt to mimic the diabetic milieu. Pretreatment of VSMC with hidrosmin dose-dependently reduced the expression of chemokines, cytokines and pro-oxidant genes induced by hyperglycemia and had a reverse effect on antioxidant genes (Figure 6a,b). At the doses employed, hidrosmin did not cause any significant reduction in cell viability, as determined by MTT assay (Supplementary Figure S2).

In another set of experiments, we investigated whether hidrosmin can stimulate eNOS activity and NO release in human endothelial cells (HMEC-1). As shown in Figure 6c, hidrosmin time-dependently induced eNOS phosphorylation at Ser1177, with maximal effect at 90 min. Consistently, we observed that hidrosmin increased endothelial NO production in a dose- and time-dependent manner (Figure 6d). 

Together, these results suggest that the beneficial effect of hidrosmin at the vascular level might be mediated, at least in part, by anti-inflammatory and antioxidant mechanisms together with NO production in vascular cells.

### 3.5. Hidrosmin Treatment Does Not Affect the Structure and Function of Hearts from Diabetic Mice

Considering that patients with T1D and T2D have an elevated risk for cardiovascular disease including cardiomyopathy, the impact of anti-diabetic agents on cardiovascular morbidity and mortality is of paramount importance. A large array of information exists about the potentially beneficial or deleterious effects of those drugs [40]. In 2018, the US Food and Drug Administration published a guidance requiring that all new drugs and biologics for glycemic control in T2D patients should demonstrate cardiovascular safety [41]. Although, this view has recently been challenged by the Endocrinologic and Metabolic Drugs Advisory Committee claiming that it is time to review this requirement for drug approval for T2D; at the preclinical level, the cardiovascular safety of new drugs should be tested. 

**Figure 7:**
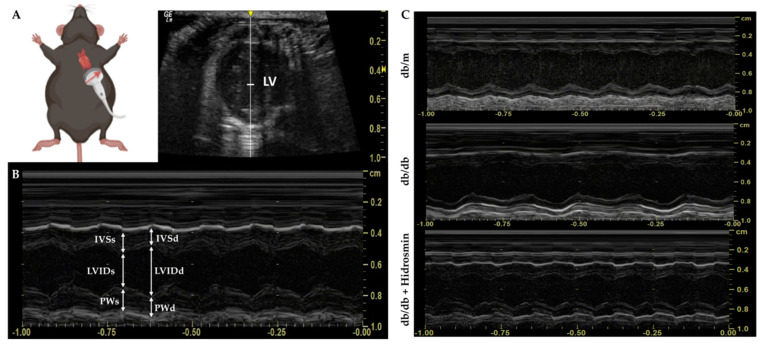
Schematic and representative views in echocardiographic assessment. (**A**) After depilation of the thoracic area, anesthetic induction with isoflurane is performed for echocardiographic evaluation. The mouse is kept in the supine position. The legs are clamped to the heating platform while the operator positions and directs the probe for the LV short-axis view. The red arrow shows the long axis of the transducer, as well as the location of the orientation notch on all transducers to guide the operator. (**B**) The use of M-mode allows assessment of systolic LV function by obtaining thicknesses of the interventricular septum, LV posterior wall and LV internal dimensions in both diastole and systole. (**C**) Representative image of sequences (at least 3 s per animal) obtained in non-diabetic (db/m), untreated diabetic (db/db) and hidrosmin-treated (db/db + Hidrosmin) mice. Abbreviations: IVS, interventricular septum; LVID, LV internal dimension; PW, LV posterior wall. All thicknesses in diastole (d) and systole (s).

Because the BKS db/db model of diabesity is a powerful preclinical tool for the study of endothelial dysfunction and diabetic cardiomyopathy with preserved ejection fraction [42,43], we examined in these mice the cardiac effect of hidrosmin by echocardiography, measuring left ventricular (LV) short-axis view (M-mode) and ventricular dimensions and thicknesses to assess systolic LV function (Figure 7). 

The results showed rather similar information to that described in non-diabetic mice (db/m) and diabetic mice (db/db), with mean ejection fractions above 70% and fractional shortening greater than 30%. Unexpectedly, LV mass corrected by body weight showed a significant reduction in diabetic mice (with and without hidrosmin) in relation to non-diabetic mice. In turn, diabetic mice treated with hidrosmin showed no changes in systolic cardiovascular function after 16 weeks of treatment compared to diabetic littermates (Table 3). These results demonstrate that hidrosmin treatment in diabetic BKS db/db mice is cardiosafe, without altering LV function and metabolic markers.

## 4. Discussion

In this paper, we show that the synthetic flavonoid hidrosmin possesses vasculoprotective effects in diabetic mice by improving endothelial function and reducing atherosclerotic plaque formation. Mechanistically, these actions are mediated by activation of eNOS and NO release, downregulation of inflammatory, oxidative stress and cellular senescence pathways and induction of antioxidant response. 

In the genesis and development of the atherosclerotic process during diabetes, the hyperglycemic milieu is one of the main accelerators of lesion progression. Hyperglycemia also modifies the NO bioavailability and eNOS uncoupling, responsible factors of the vascular reactivity changes and endothelial dysfunction [4,5,10]. The beneficial effects of different flavonoids in cardiovascular complications of diabetes have been studied in experimental models of T1D and T2D [18,23,25,44]. Micronized purified flavonoid fractions containing phlebotonic drugs such as diosmin and hidrosmin have been used in the treatment of chronic venous insufficiency likely because of its ability to ameliorate venous tone and contractility [21,45]. However, to our knowledge this is the first study evaluating in relevant preclinical models the effects of hidrosmin on the vascular dysfunction associated with hyperglycemia. In agreement with previous studies in diabetic mice [11,37,38,39], we observed that aorta and mesenteric arteries from db/db mice exhibited impaired endothelial function evidenced by reduced acetylcholine-induced endothelium-dependent vasorelaxation and aortic hypercontractility. Remarkably, we found that hidrosmin therapy improved endothelial dysfunction in aorta and mesenteric arteries of db/db mice and reduced vascular contractility to phenylephrine in mesenteric arteries but not in aorta. Hidrosmin was also able to induce vascular relaxation that was inhibited by L-NAME and to stimulate in vitro the activation/phosphorylation of eNOS and NO generation in endothelial cells. Our data therefore suggest that the benefits of hidrosmin treatment on vascular function are mediated by NO, which is in agreement with the reported ability of different flavonoids to increase NO production [46,47,48].

T2D has been associated with vascular remodeling and progressive increase in vascular stiffness [49]. While the effects of T2D in small arteries function have been well established, the type of vascular remodeling observed in animal models or patients is controversial. Subcutaneous fat small resistance arteries from T2D patients showed hypertrophic remodeling and decreased fibrosis [50]. However, resistance arteries of hypertensive patients with pre-existing T2D presented eutrophic remodeling and increased fibrosis [51]. In small resistance arteries of db/db mice, outward hypertrophic remodeling [52], inward hypertrophic remodeling [37,53] or similar vascular structures have been found [9], likely due to the different backgrounds, ages or diets. Herein, we have observed that mesenteric arteries from db/db mice in BKS susceptibility strain presented hypertrophic outward remodeling that was not modified by treatment with hidrosmin. Similar results were observed in aorta.

The beneficial effects of hidrosmin on atherosclerotic lesions were very remarkable. Since the db/db diabetic mice do not develop marked atherosclerosis lesions at the conditions and time period studied, we employed the STZ-induced diabetic ApoE KO mice, a well-established mouse model of accelerated atherosclerosis driven by combined hyperglycemia and hyperlipidemia that resembles human atherosclerotic lesions [31,32]. In brief, the treatment with hidrosmin markedly decreased the size, extent and composition of atheroma plaques. Of particular interest was the impact of hidrosmin on several histological features of plaque stability, as noted by lower lipid deposition and higher collagen content and therefore a significantly higher collagen-to-lipid ratio. The increased content of VSMC observed in plaques of hidrosmin-treated mice is also compatible with a lower plaque vulnerability. Furthermore, the Stary classification confirmed that hidrosmin-treated mice presented less severe atherosclerotic lesions (grade G4) than untreated diabetic mice. 

Our results indicate that diabetic ApoE KO mice respond better to hidrosmin treatment than db/db mice in terms of biochemical parameters, probably because the genetic background and metabolic alterations of these models have distinct consequences on the susceptibility to develop dyslipidemia, obesity or atherosclerosis [31]. Consistent with our previous study [30], oral administration of hidrosmin in diabetic ApoE KO mice did not affect hyperglycemia but partially reduced serum levels of total cholesterol and triglycerides. However, the relatively modest effect on these parameters could not explain the marked reduction in atheroma burden that could be due to multiple vasculoprotective actions of the flavonoid. In fact, hidrosmin-treated mice exhibited a reduced content of macrophages and T cells in atheroma plaques and downregulated expression of pro-inflammatory cytokines involved in leukocyte recruitment and activation. Hidrosmin also restored the expression of genes involved in redox balance and prevented the accumulation of oxidative DNA damage biomarker in aorta of diabetic mice. Concurrently, hidrosmin attenuated the expression of p16, a premature senescence marker induced by inflammation and oxidative stress [54] and TERT, a telomerase subunit activated during atherogenesis and a feed-forward loop for inflammatory gene expression [55]. On the cellular level, we demonstrated a dose-dependent downregulation of cytokines, chemokines and NOX isoforms by hidrosmin in VSMC exposed to hyperglycemia, as well as SOD1 and catalase upregulation, thus confirming its anti-inflammatory and antioxidant action in the diabetes context. 

Many studies of flavonoid bioactivity have reported the complexity of the mechanism of action and the diversity of cellular targets. Several dietary polyphenols are able to improve insulin sensitivity and modulate energy metabolism by inducing sirtuin 1 gene expression and AMP-activated protein kinase phosphorylation and to downregulate key inflammatory pathways, such as protein kinase C, inducible NOS, cyclooxygenase-2, mitogen-activated protein kinase and different transcription factors. In diabetic animals, epigallocatechin gallate regulates insulin receptor by inhibiting tyrosine phosphatases, catechin enhances phosphatidylinositol-3-kinase and eNOS signaling system, and aspalathin downregulates apoptosis by suppressing nuclear factor-κB (NF-κB) and target gene expression [56]. Our recent study in diabetic nephropathy showed that hidrosmin actions are mediated by the concerted inhibition of NF-κB and Signal Transducer and Activator of Transcription 3 (STAT3) in renal cells [30], which is in line with the normalization of renal NF-κB activity reported for diosmin in diabetic rats [57]. Based on this, we propose that the molecular mechanism of hidrosmin bioactivity in vascular cells may involve the regulation of NF-κB and STAT, key transcription factors underpinning inflammatory, oxidative and senescent processes in diabetes. Future studies are warranted to understand the signaling cascades behind the vascular actions of hidrosmin.

Previous reports in rodent models of diabetes have pointed out the beneficial effect of natural flavonoid diosmin and synthetic flavonoid hidrosmin on diabetic nephropathy and cardiomyopathy by reducing hyperglycemia-mediated oxidative stress and inflammation [23,30,57]. However, to the best of our knowledge, this study combining two complementary experimental models is the first to demonstrate the positive impact of hidrosmin on vascular function in aorta and mesenteric arteries of db/db mice and atherosclerotic plaques of diabetic ApoE KO mice, which are corroborated in vitro in endothelial cells and hyperglycemia-exposed VSMC. We also provide evidence of the beneficial metabolic effects and cardiovascular safety of hidrosmin in the context of T1D and T2D.

In summary, our preclinical results illustrate that the synthetic flavonoid hidrosmin can limit endothelial dysfunction and atherosclerotic progression in diabetes. Although further studies are needed to evaluate the effectiveness of this therapy, hidrosmin could have a potential role as a co-adjuvant therapy for the chronic macrovascular complications of diabetes by reducing the cardiovascular residual risk.

## Figures and Tables

**Table 1:** Metabolic and biochemical parameters in non-diabetic (db/m), untreated diabetic (db/db) and hidrosmin-treated diabetic (db/db + H) mice. Data are expressed as mean ± SEM of *n* = 10 animals per group. * *p* < 0.05 vs. db/m analyzed separately by Mann–Whitney U test. Abbreviations: HDL-C, HDL cholesterol; LDL-C, LDL cholesterol; ASAT, aspartate aminotransferase; ALAT, alanine aminotransferase; BUN, blood urea nitrogen; UD, undetectable.

	Db/m	db/db	db/db + H
Blood Glucose (mg/dL)	139.3 ± 5.1	396.4 ± 25.9 *	385.3 ± 26.0
Body Weight (g)	32.2 ± 0.46	52.8 ± 1.4 *	50.4 ± 1.9
Triglycerides (mg/dL)	84.3 ± 11.5	94.3 ± 13.5	91.8 ± 11.3
Total Cholesterol (mg/dL)	88.6 ± 2.1	171.6 ± 10.2 *	165.6 ± 7.6
LDL-C (mg/dL)	UD	26.3 ± 3.3	24.3 ± 2.7
HDL-C (mg/dL)	76.3 ± 1.6	128.5 ± 6.4 *	125.9 ± 4.7
ASAT (UI/L)	111.2 ± 31.2	135.8 ± 11.1	125.8 ± 8.9
ALAT (UI/L)	39.3 ± 4.1	150.4 ± 18.0 *	148.6 ± 17.1
Urea (mg/dL)	36.6 ± 1.3	42.0 ± 2.4	43.0 ± 1.3
BUN (mg/dL)	17.3 ± 0.6	19.6 ± 1.1	20.0 ± 0.6

**Table 2:** Effect of hidrosmin on metabolic parameters in diabetic ApoE KO mice. Data are expressed as mean ± SEM of each group (untreated (D), *n* = 7; hidrosmin treatment (D + H), *n* = 11). * *p* < 0.05 vs. D group by Mann–Whitney U test. Abbreviations: LDL-C, LDL cholesterol; HDL-C, HDL cholesterol.

	D	D + H
Body Weight (g)	21.7 ± 0.8	21 ± 0.9
Blood Glucose (mg/dL)	489.9 ± 27.8	418.3 ± 34.0
Triglycerides (mg/dL)	241.1 ± 51.2	129.2 ± 17.7 *
Total Cholesterol (mg/dL)	928.3 ± 82.4	669.25 ± 82.7 *
LDL-C (mg/dL)	694.5 ± 116.7	565.8 ± 82.5
HDL-C (mg/dL)	95.7 ± 6.8	83.9 ± 7.3

**Table 3:** Echocardiographic assessment and parameters evaluated in db/db mouse model. Data are expressed as mean ± SEM of each group (db/m, *n* = 8; db/db, *n* = 8; db/db + H, *n* = 8). * *p* < 0.05 vs. db/m analyzed separately by Mann–Whitney U test. Abbreviations: IVS, interventricular septum; LVID, LV internal dimension; PW, LV posterior wall. All thicknesses in diastole (d) and systole (s). BW, body weight correction; EF, LV ejection fraction; FS, LV fractional shortening; PWT, LV posterior wall thickening.

Variables	db/m	db/db	db/db + H
IVSd (μm)	102.4 ±4.3	115.4 ± 15.2	109.6 ± 15.1
LVIDd (μm)	379.5 ± 4.8	336.2 ± 46.6	336.3 ± 37.1
PWd (μm)	99.0 ± 2.4	106.2 ± 12.9	94.2 ± 11.8
IVSs (μm)	144.8 ± 2.7	171.9 ±18.9	165.8 ± 18.0
LVIDs (μm)	260.0 ± 4.5	222.4 ± 36.7	219.2 ± 25.3
PWs (μm)	138.6 ±2.4	137.1 ± 13.9	120.4 ± 16.9
LV mass (μg)	148.6 ± 4.7	146.4 ± 19.5	125.3 ± 16.6
LV mass/BW (μg/g)	5.0 ± 0.2	2.6 ± 0.4 *	2.4 ± 0.3 *
EF (%)	67.1 ± 2.4	71.2 ± 9.9	72.1 ± 10.2
FS (%)	31.3 ± 1.6	34.2 ± 5.0	35.4 ± 5.4
PWT (%)	40.5 ± 5.1	28.8 ± 7.4	29.2 ± 8.3

## Data Availability

Data is contained within the article and Supplementary Material.

## Supplementary Materials

The following supporting information can be downloaded at: https://www.mdpi.com/article/10.3390/antiox11122499/s1, Table S1: Mouse primers used for real-time qPCR; Table S2: Summary of the measurements of vascular reactivity in the db/db mouse model; Figure S1: Follow-up of metabolic parameters in diabetic ApoE KO mice; Figure S2: Hidrosmin does not affect viability of VSMC.
