# Supplementary material for: The Synthetic Flavonoid Hidrosmin Improves Endothelial Dysfunction and Atherosclerotic Lesions in Diabetic Mice
Source: Antioxidants (Basel). 2022 Dec 19;11(12):2499. doi: 10.3390/antiox11122499 (PMC9774734; doi:10.3390/antiox11122499)
Supplement: Supplementary file 1 [file antioxidants-11-02499-s001.zip › antioxidants-2010469-supplementary.pdf]

**Supplementary Material**

The synthetic flavonoid Hidrosmin improves endothelial dysfunction and atherosclerotic lesions in diabetic mice. Jimenez=Castilla et al.

| SYBR Green Primers | | |
|---|---|---|
| **Gene (ID)** | **Forward** | **Reverse** |
| *Nox1* (237038) | CCAACAGGCCATGGATGGAT | CACTCCAGTAAGCCAGCAA |
| *Nox4* (50490) | CCCTCCTGGCTGCATTAGTC | AACCCTCGAGGCAAAGATCC |
| *18S rRNA* (19791) | CCGTCGTAGTTCCGACCATAA | CAGCTTTGCAACCATACTCCC |
| **Taqman Primers** | | |
| **Gene** | **Assay ID** | |
| *Ccl2* | Mm00441242_m1 | |
| *Ccl5* | Mm01302428_m1 | |
| *Il1-β* | Mm00434228_m1 | |
| *Tnfα* | Mm00443258_m1 | |
| *Sod1* | Mm01344233_g1 | |
| *Cat* | Mm00437992_m1 | |
| *Tert* | Mm00436934_m1 | |
| *p16* | Mm00494449_m1 | |
| *18s rRNA* | 4310893E | |

**Supplementary Table S1.** Mouse primers used for real-time qPCR.

| | Response | | db/m | db/db | db/db +H |
|---|---|---|---|---|---|
| AORTA | Phe | EC50 (mM) | 3.04±2.64 | 0.04±0.006 * | 0.04±0.004* |
| | | Rmax (mN/mm) | 5.43±0.92 | 12.09±1.19* | 12.76±0.95 * |
| | Ach | EC50 (mM) | 0.14±0.03 | 0.41±0.19 | 0.07±0.02 # |
| | | Rmax (%) | 67.35±2.13 | 40.57±5.83 * | 51.6±6.23 |
| | DEA-NO | EC50 (mM) | 0.69±0.06 | 0.63±0.08 | 0.77±0.22 |
| | | Rmax (%) | 96.03±2.6 | 97.51±0.7 | 96.61±0.9 |
| MRAs | Phe | EC50 (mM) | 0.18±0.02 | 0.15±0.02 | 0.34±0.8 # |
| | | Rmax (mN/mm) | 2±0.16 | 1.8±0.17 | 1.6±0.26 |
| | Ach | EC50 (mM) | 0.029±0.009 | 0.472±0.231 * | 0.013±0.003 # |
| | | Rmax (%) | 73.34±8.5 | 64.98±10 | 74.51±7.8 |
| | DEA-NO | EC50 (mM) | 0.6±0.4 | 0.19±0.06 | 0.27±0.08 |
| | | Rmax (%) | 94.37± 1.9 | 96.67± 0.57 | 95.88 ±0.88 |

**Supplementary Table S2.** Summary of the measurements of vascular reactivity in the db/db mouse model. Values of half maximal effective concentration ($EC_{50}$) and maximum response (Rmax) to phenylephrine (Phe), Acetilcholine (Ach) and diethylamine NONOate (DEA-NO) in aorta and mesenteric resistance arteries (MRAs) segments from non-diabetic (db/m), untreated diabetic (db/db) and hidrosmin-treated diabetic (db/db+H) mice. *$p$<0.05 vs. db/m and #$p$<0.05 vs. db/db by Kruskal-Wallis test followed by Dunn's post-test.

**a**

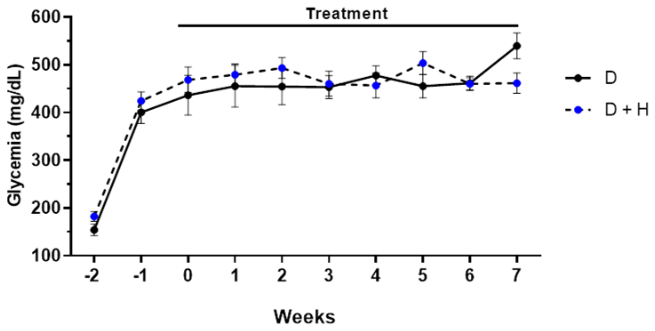


**b**

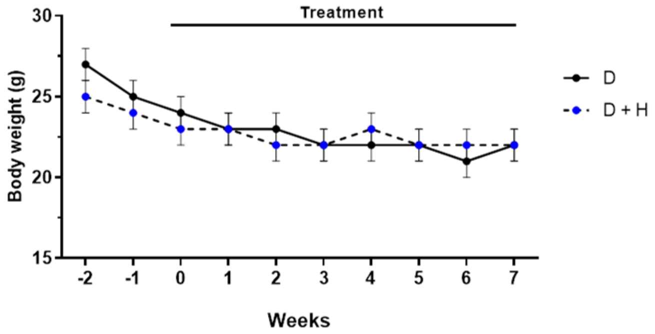


**Supplementary Figure S1:** Follow-up of metabolic parameters in diabetic ApoE KO mice. Evolution of glycemia (**a**) and body weight (**b**) of studied groups (untreated, D; hidrosmin treatment, D+H) after diabetes induction with STZ and during the treatment period. The graphs represent the mean ± SEM of each group (D, n=7; D+H, n=11).

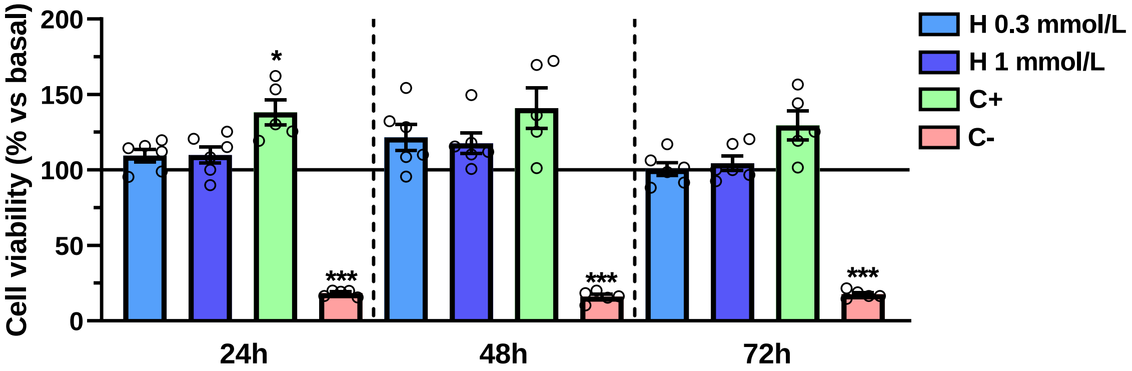


**Supplementary Figure S2:** Hidrosmin does not affect viability of VSMC. MTT viability assay in primary mouse VSMC incubated for 24, 48 and 72 h in medium with 0.5% FBS containing hidrosmin (H, 0.3 and 1 mmol/L), 20% FBS (positive control, C+) and 10% DMSO (negative control, C-). Bars represent the mean ± SEM of 5-6 experiments analyzed in triplicate. *$p$<0.05 and ***$p$<0.001 vs. basal conditions (black line).
